# Environmental surveillance: harnessing environmental sample data to protect animal health

**DOI:** 10.3389/fvets.2026.1965480

**Published:** 2026-09-29

**Authors:** Bayyppa Gundallahalli Manjunatha Reddy, Uzma Jabeen, Hinal Kharva, Muthannan Andavar Ramakrishnan

**Affiliations:** 1 ICAR-National Institute of Veterinary Epidemiology and Disease Informatics, Bengaluru, Karnataka, India; 2 National Centre for Biological Sciences, Tata Institute of Fundamental Research, Bangalore, Karnataka, India; 3 ICAR – Indian Veterinary Research Institute, Bengaluru, Karnataka, India

**Keywords:** animal disease surveillance, environmental surveillance, molecular detection, non-invasive surveillance, One Health, wastewater-based epidemiology

## Abstract

Infectious diseases in domestic animals—including those with zoonotic potential—impose a substantial global burden on food security, livelihoods, and public health. Environmental surveillance (ES) offers a critical complement to conventional animal-level diagnostics by detecting pathogens or their genetic signatures directly in the matrices through which they persist and spread: water, soil, air, manure, fomites, and arthropod vectors. This review synthesizes evidence across three analytical dimensions—matrix suitability, detection method performance, and wastewater-based epidemiology (WBE)—for 15 viral, bacterial, and parasitological diseases of domestic animals. Key findings reveal that matrix choice profoundly governs detection sensitivity: surface water and wetland sediments support infectious avian influenza virus isolation; pooled environmental feces and slurry outperform individual animal sampling for *Mycobacterium avium subsp. paratuberculosis* (MAP); and electrostatic dust cloths enable simultaneous detection of peste-des-petits ruminants virus (PPRV) and capripoxviruses at livestock markets. Molecular methods, principally quantitative RT-PCR and droplet digital PCR, consistently outperform conventional culture in sensitivity and turnaround time, though viability-linked assays remain an important unresolved need. WBE is increasingly applied beyond human pathogens: piggery effluent surveillance for Japanese encephalitis virus and wastewater metagenomics for antimicrobial resistance exemplify its veterinary potential. Interpretive limitations persist, including matrix-driven inhibition, absence of harmonized protocols, and the challenge of equating nucleic-acid signals with infectious risk. Realizing the full promise of ES within a One Health framework requires standardized, field-validated sampling workflows, viability-linked molecular assays, and formal integration of environmental signals into routine veterinary surveillance and outbreak-response systems.

## 1 Introduction

Environmental surveillance (ES) is an increasingly indispensable component of infectious disease monitoring in domestic animal populations. Rather than relying exclusively on clinical presentation or individual animal testing—approaches that are inherently reactive and epidemiologically incomplete—ES interrogates the matrices through which pathogens are shed, persist, and transmitted. By doing so, it can detect pathogen circulation ahead of clinical case accrual, characterize transmission hotspots, and generate population-level risk signals that individual animal-based surveillance structurally cannot. This capacity is particularly valuable under field conditions where direct sampling is logistically constrained—migratory bird flocks, wild boar populations, informal livestock markets, and free-ranging ruminants all present practical barriers to representative animal-level sampling that ES can partially circumvent.

In practice, ES encompasses the systematic collection and analysis of water, air, soil, bedding, manure, fomites, arthropod vectors, and other environmental matrices from farms, markets, water bodies, and shared wildlife–livestock interfaces (1, 2). These samples can reveal pathogen presence, environmental persistence half-lives, genetic diversity, and shedding dynamics—information that collectively sharpens our understanding of source attribution and transmission route characterization. The advent of environmental DNA (eDNA) and environmental RNA (eRNA) methodologies, combined with quantitative PCR (qPCR), droplet digital PCR (ddPCR), and metagenomic sequencing, has dramatically extended the analytical sensitivity and breadth of ES, enabling detection of pathogens at concentrations that culture-based and antigen detection approaches routinely fail to capture.

Expanding the ES framework substantially, wastewater-based epidemiology (WBE) utilizes municipal and on-farm wastewater as aggregated, non-invasive sentinels for pathogen circulation at population scale (Figure 1). Originally pioneered for poliovirus and accelerated during the COVID-19 pandemic, WBE is now demonstrating veterinary relevance. For example, piggery effluent surveillance has successfully detected Japanese encephalitis virus (JEV) RNA during active outbreaks (3). Furthermore, metagenomic WBE can profile antimicrobial resistance gene (ARG) reservoirs across entire livestock production catchments (4, 5). Complementarily, remote sensing and geospatial analysis—mapping vector breeding habitats, wetland connectivity, and wildlife movement corridors—provide ecological context that transforms discrete environmental detections into spatially coherent risk maps (6, 7). To systematically evaluate the state of knowledge and operational utility of ES, this review synthesizes evidence across 15 major infectious diseases of domestic livestock and poultry, categorized into three etiologic groups: (1) viral diseases (*n* = 7): Foot-and-mouth disease (FMD), Peste des petits ruminants (PPR), Lumpy skin disease (LSD), Avian influenza (AI), Newcastle disease (ND), African swine fever (ASF), and Japanese encephalitis (JE); (2) bacterial diseases (*n* = 4): Salmonellosis (*Salmonella enterica*), Paratuberculosis / Johne’s disease (*Mycobacterium avium subsp. paratuberculosis*, MAP), Leptospirosis (pathogenic *Leptospira* spp.), and Bovine tuberculosis (*Mycobacterium bovis*, bTB); and (3) parasitological diseases (*n* = 4): Coccidiosis (*Eimeria* spp.), Fasciolosis (*Fasciola hepatica* and *F. gigantica*), Babesiosis (*Babesia* spp.), and Theileriosis (*Theileria* spp.). This review synthesizes these dimensions across 15 diseases of domestic animals, evaluating ES through three analytical lenses: (i) matrix suitability and pathogen survival, (ii) detection method performance under field conditions, and (iii) the emerging contribution of WBE and non-invasive sampling. It identifies where ES provides its most distinctive epidemiological utility, and articulates the operational and technical challenges that currently constrain routine implementation.

**Figure 1:**
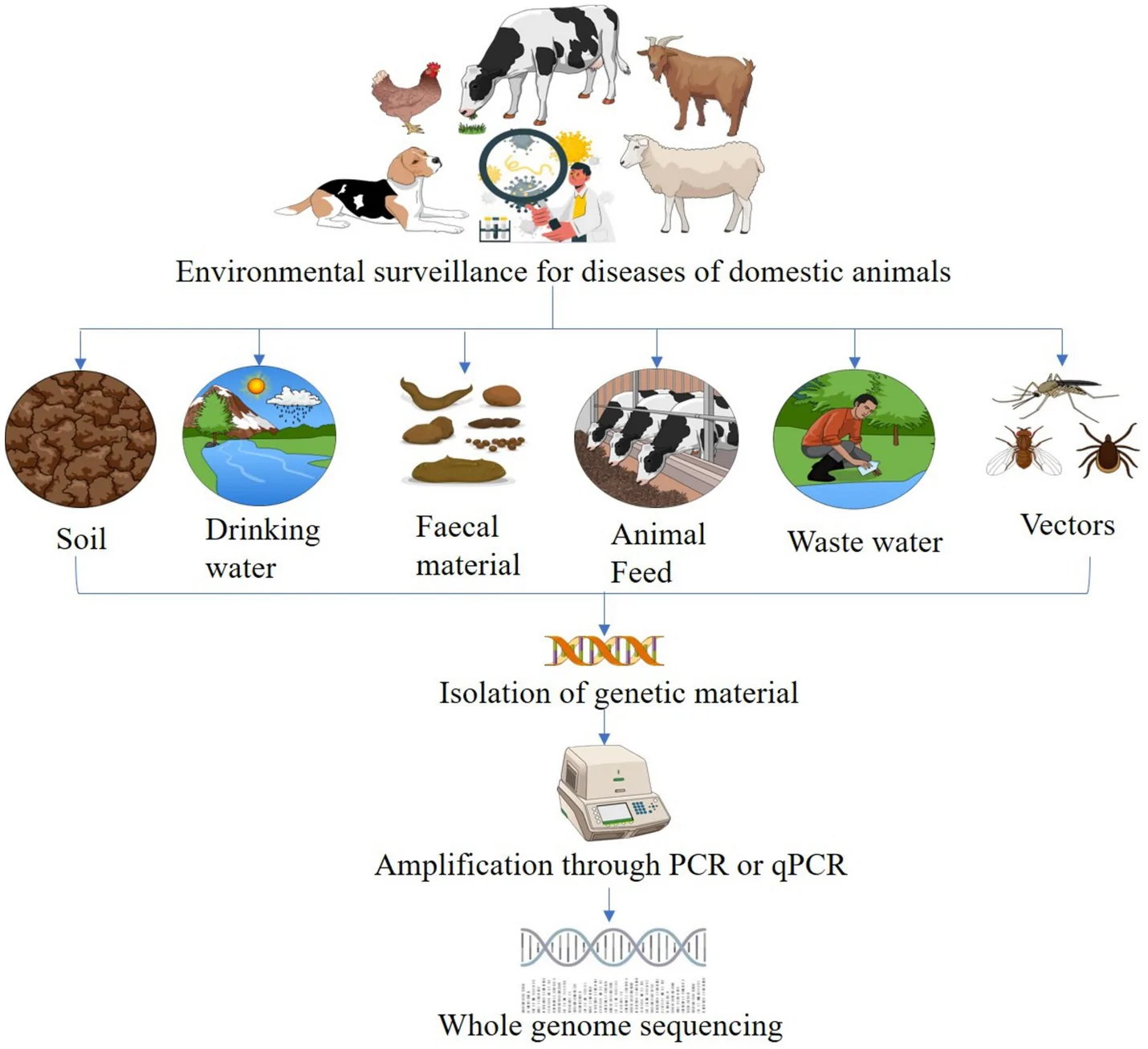
A conceptual summary of environmental monitoring for animal health, illustrating key sample matrices (water, air, soil, manure, fomites, and arthropod vectors) and laboratory detection workflows utilized for early warning, source attribution, and risk-informed veterinary decisions (infographics created using vecteezy.com, freepik.com, and sciencefigures.org). eDNA, environmental DNA; eRNA, environmental RNA; qPCR, quantitative PCR; ddPCR, droplet digital PCR; WBE, wastewater-based epidemiology; NGS, next-generation sequencing.

## 2 Methodologies of environmental surveillance

The efficacy of ES hinges on the appropriate selection and application of various methodologies for sample collection, pathogen detection, and data analysis.

### 2.1 Sample collection

Environmental samples can be diverse, reflecting the various routes of pathogen shedding and persistence in the environment. Common sample types include:

- Water and effluent samples: Collected from drinking troughs, surface waters, drainage run-off, lagoons, and municipal/on-farm effluent streams to detect waterborne viruses and enteric bacteria (5). Operational protocols typically require 500 mL to 5 L volumes for surface waters (concentrated via dead-end ultrafiltration or electronegative membrane adsorption) and 100 mL to 1 L for high-turbidity slurry or wastewater effluents (processed via skimmed-milk flocculation or centrifugal ultrafiltration). 24-h composite sampling is recommended for effluent streams to account for diurnal shedding variations, accompanied by trip and field blank quality controls.
- Air samples: Collected using liquid impingers, solid impactors, or cyclone air samplers to detect aerosolized respiratory pathogens in confined animal housing, live bird markets, and farm perimeters ((8, 9)). Standardized protocols employ personal or stationary liquid impingers (e.g., operating at 12.5 L/min for 30–60 min into collection fluids supplemented with viral transport medium and protein stabilizers) or high-volume dry cyclone samplers (50–100 L/min for 1–2 h). Field air blanks and meteorological records (relative humidity, temperature, airflow velocity) are mandatory for quantitative comparability.
- Soil and bedding samples: Relevant for environmentally robust pathogens such as bacterial spores (*Bacillus anthracis*), mycobacteria, and protozoan oocysts that persist in soil microhabitats or barn bedding (10). Protocols necessitate core sampling across multiple high-traffic points (minimum 5–10 sub-samples per pen or pasture sector, pooled to 100–250 g), with immediate transport at 4 °C, desiccation prevention, and mechanical bead-beating cell disruption to overcome soil particle adsorption.
- Fomite and surface samples: Swabs from surfaces, equipment, vehicles, animal crates, or footwear that facilitate indirect transmission (11). Standardized protocols use pre-moistened macrofoam or synthetic swabs (wetted with buffered peptone water, PBS containing 0.1% Tween-20, or viral transport medium) rubbed across defined surface areas (100–500 cm^2^ per surface). For large-area poultry floor monitoring, paired boot-sock swabs walked over a minimum of 100 m traverse provide operationally validated flock-level sensitivity.
- Manure and slurry samples: Pooled composite samples from animal waste channels, slurry pits, or manure heaps offering aggregated herd-level shedding signals (12). Representative protocols require 10–20 sub-aliquots collected across dung channels or holding lagoons (total 50–100 g composite), followed by thorough homogenization, enzymatic/mechanical lysis, and the incorporation of internal amplification controls to detect potent PCR inhibitors (bile salts, polyphenols, and humic compounds).
- Dust samples: Accumulations of settled or airborne dust in livestock facilities contain desiccation-resistant pathogen nucleic acids, providing a cumulative measure of shedding over days to weeks (7). Standardized electrostatic dust wipes (20 × 20 cm cloths sampled over 1–5 m^2^ horizontal surfaces) or passive settling plates deployed for 7–14 days allow low-cost, non-invasive herd-level surveillance at animal aggregation points and live markets.

The selection of sample type is influenced by the pathogen’s epidemiological traits, its ability to persist in various environments, and the goals of the surveillance effort. For instance, air sampling is typically most effective for respiratory pathogens, while manure or wastewater is often more appropriate for enteric pathogens that are excreted at the group level.

Ensuring proper sample preservation and transport is crucial for accurate interpretation. Handling procedures specific to the matrix, such as maintaining temperature control (cold chain at 4 °C for immediate processing or −80 °C for biobanking), rapid transport, the addition of antimicrobial/antifungal cocktails to culture media, and the prevention of repeated freeze–thaw cycles, must be strictly enforced. Comprehensive quality assurance requires the routine inclusion of field trip blanks (to monitor collection and transport contamination), negative extraction controls, matrix-matched positive spikes (to calculate recovery efficiency), and exogenous internal amplification controls (IACs, such as bacteriophage MS2, equine arteritis virus, or phocid herpesvirus-1) spiked into every PCR reaction to detect false-negative results caused by environmental matrix inhibitors.

### 2.2 Pathogen detection methods

Once samples are collected, various laboratory techniques are employed for pathogen detection:

- Molecular Methods:
  - Polymerase Chain Reaction (PCR) and quantitative PCR (qPCR): Widely used for rapid and sensitive detection of pathogen nucleic acids (DNA/RNA). qPCR provides quantitative data on pathogen load (13).
  - Next,-Generation Sequencing (NGS) / Metagenomics: Offers broad-spectrum detection of known and unknown pathogens, enabling strain typing, antimicrobial resistance gene detection, and understanding of microbial communities (4).
  - Loop-mediated Isothermal Amplification (LAMP): A simpler, rapid, and often field-deployable molecular technique for pathogen detection (14).
- Culture-based methods: involve traditional microbiological approaches for isolating and identifying living pathogens. Although these methods are slower, they confirm the pathogens’ viability and enable further analysis, such as testing for antimicrobial susceptibility.
- Antigen Detection Assays: Less common for environmental samples but can be used for specific pathogens where robust antibody-based detection is feasible (15).
- Biosensors: are becoming valuable additions to traditional lab techniques due to their ability to facilitate quick and portable detection directly in the field. These instruments integrate a biological recognition component, such as an antibody, enzyme, or nucleic-acid probe, with a transducer that translates the biological interaction into a quantifiable electrical, optical, or thermal signal (16). Their advantage lies in their speed and suitability for field use, although it is still essential to validate their analytical performance across complex environmental matrices.

When evaluating detection methods, it is crucial to consider factors such as matrix effects, recovery efficiency, analytical sensitivity, specificity, and the difference between identifying nucleic acid and proving the presence of a viable pathogen. Quality control necessitates the use of field blanks, extraction and amplification controls, conducting tests in duplicates when possible, and clearly reporting detection limits. In practice, the selection of a method is also influenced by factors like cost, turnaround time, available infrastructure, and whether the goal of surveillance is rapid screening, confirmatory diagnosis, strain characterization, or ongoing monitoring. The relative performance of these techniques across different environmental matrices, and their practical trade-offs under field conditions, are summarized in Table 1 and revisited in the context of specific disease applications in Section 3 (Figure 2).

**Table 1:** Comparative overview of environmental pathogen detection methods.

**Method**	**Analytical sensitivity**	**Diagnostic specificity**	**Field deployability**	**Field validation status**	**Cost per sample**	**Optimal matrix**
Culture / isolation	Low–moderate (viability required)	High (definitive isolate characterization)	No (BSL facility needed)	Operationally established (regulatory reference standard)	Low–moderate	Water, soil, swabs
qPCR / RT-qPCR	High (≤10 copies/reaction)	High (target-specific primers and probes)	Limited (portable field units emerging)	Operationally Established (widely deployed in field and outbreaks)	Moderate	Water, swabs, feces, air, dust
ddPCR	Ultra-high (absolute copy quantification)	High (probe-specific)	No (centralized laboratory)	Field validated / Investigational (high-precision target quantification)	Moderate–high	Complex effluent, low-titer water, sediment
LAMP	Moderate–high (10–100 copies/reaction)	High (4–6 specific primers)	Yes (isothermal, battery-powered)	Field Validated / Point-of-Care (operational in low-resource settings)	Low	Feces, water, pen swabs, milk
NGS / Metagenomics	High (untargeted breadth)	Very high (strain typing and ARG resolution)	No (centralized sequencing platform)	Investigational / Research Phase (routine in WBE catchments)	High	Wastewater, slurry lagoons, dust traps
Antigen assays (LFA)	Low–moderate	Moderate–high	Yes (lateral flow format, 15–30 min)	Operationally established (point-of-need screening; limited ES sensitivity)	Low	Feces, high-titer tissue swabs
Biosensors	Moderate (matrix-dependent)	Moderate–high	Yes (portable microdevices, real-time)	Investigational / Prototype phase (matrix biofouling is a field barrier)	Moderate	Clarified water, drainage run-off

qPCR, quantitative PCR; RT-qPCR, reverse transcription-quantitative PCR; ddPCR, droplet digital PCR; LAMP, loop-mediated isothermal amplification; NGS, next-generation sequencing; LFA, lateral flow assay; BSL, biosafety level; ARG, antimicrobial resistance gene; ES, environmental surveillance; WBE, wastewater-based epidemiology. Field validation status classifications: Operationally Established (standardized official method); Field Validated (tested and verified in real-world field/outbreak settings); Investigational / Prototype (promising laboratory/pilot evaluations).

**Figure 2:**
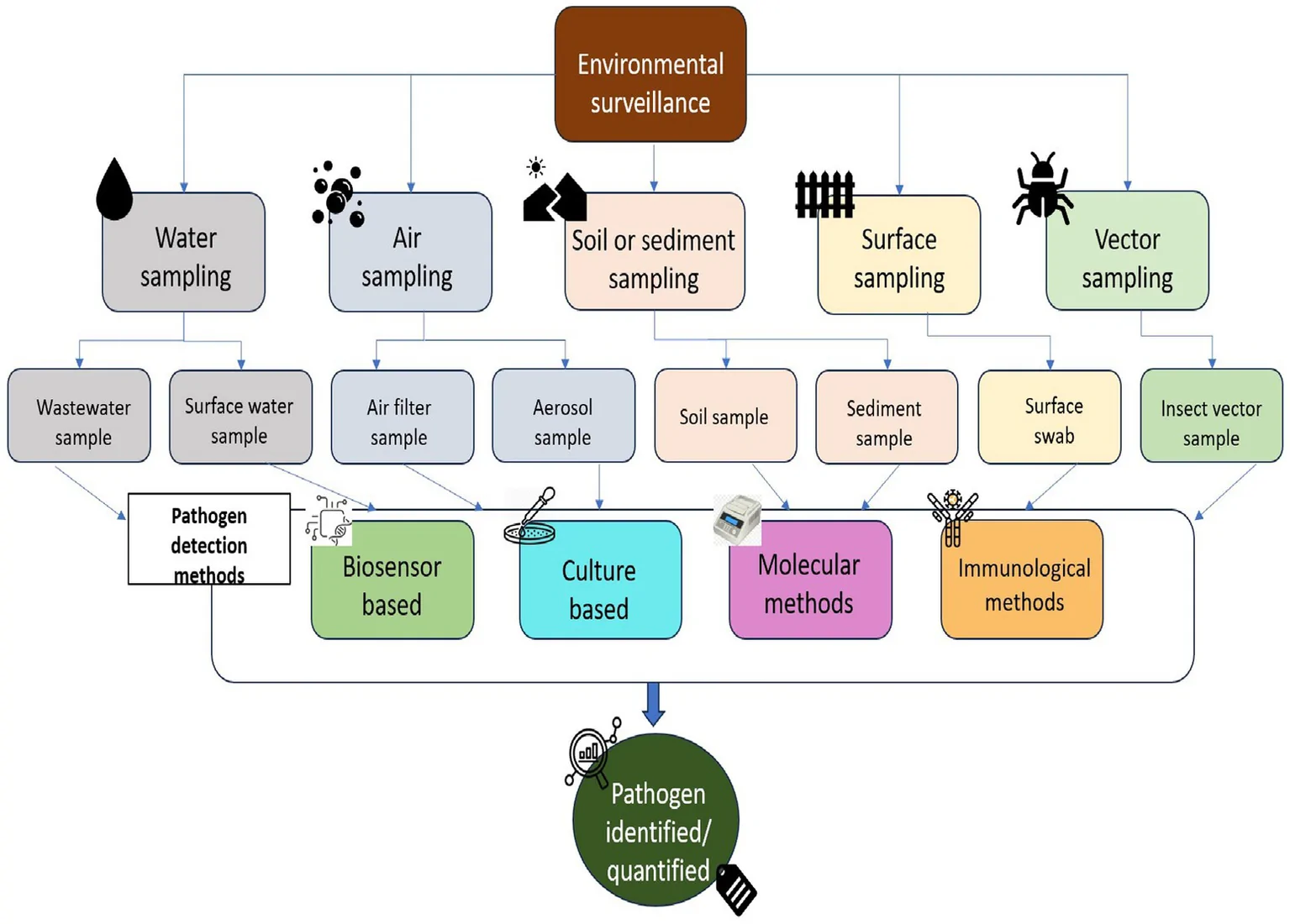
An illustration summarizing the environmental surveillance methodology.

### 2.3 Wastewater-based epidemiology in veterinary contexts

Wastewater-based epidemiology (WBE) has emerged as one of the most consequential methodological advances in population-level pathogen surveillance. Its foundational logic is straightforward: infected individuals—whether human or animal—shed pathogens or their nucleic acids in feces, urine, and respiratory secretions, which enter wastewater streams and can be detected before clinical case counts reflect true transmission intensity. During the COVID-19 pandemic, WBE demonstrated a capacity to anticipate clinical surges by 7–14 days in urban catchments (4, 5), establishing a blueprint now being adapted to veterinary and One Health applications.

In domestic animal contexts, the most compelling application of WBE to date involves on-farm effluent surveillance for Japanese encephalitis virus. Ahmed et al. (3) demonstrated that JEV RNA is consistently detectable in piggery effluent during active transmission seasons, offering a herd-level signal that bypasses the logistical complexity of individual pig sampling in large intensive operations. The complementarity with mosquito-trap surveillance is striking: while vector trapping captures transmission pressure in the broader environment, piggery WBE reflects viral amplification within the key vertebrate amplifier host, potentially enabling earlier intervention. Similarly, municipal wastewater surveillance detected JEV RNA during an acute disease outbreak in Australia, underscoring the broader catchment-level sensitivity of this approach (3). A concrete operational blueprint for veterinary WBE is provided by wastewater monitoring for JEV in intensive pig production catchments. In this integrated surveillance framework: (1) routine weekly effluent composite samples are collected from piggery drainage channels and effluent ponds; (2) rapid RT-qPCR screening quantifies JEV RNA load relative to an endogenous control; (3) crossing an actionable threshold (e.g., progressive cycle threshold decline or >10^3 viral genome copies/L) immediately triggers a multi-agency tiered response: (a) targeted vector surveillance intensification by deploying CO2-baited light traps within a 3–5 km buffer zone to determine *Culex tritaeniorhynchus* / *Culex annulirostris* abundance and vector infection rates; (b) immediate vector abatement through larvicide application in effluent ponds and surrounding breeding waters, accompanied by barrier thermal fogging with adulticides; (c) herd health interventions, including piggery movement standstill orders, vector screening of breeding stock, and booster vaccination where authorized; and (d) public health notifications to local health authorities to issue mosquito avoidance advisories, enforce personal protective equipment for farm personnel, and prioritize human JEV vaccination in surrounding communities. This operational continuum exemplifies how environmental pathogen detection in animal waste streams directly informs upstream vector control and prevents downstream zoonotic spillover.

Beyond specific viral targets, metagenomic WBE offers unbiased pathogen and antimicrobial resistance gene (ARG) surveillance without *a priori* target selection. Brumfield *et al.* (4) demonstrated that metatranscriptomic sequencing of wastewater captures not only SARS-CoV-2 but co-circulating bacterial and parasitic agents, viral diversity indices, and ARG profiles—collectively providing a rich epidemiological landscape. For veterinary surveillance, this is particularly relevant to intensive livestock production systems, where dense animal housing, prophylactic antimicrobial use, and high fecal biomass create wastewater streams rich in both pathogens and resistance determinants. Nguyen *et al.* (12) identified a broad array of potential bacterial pathogens and ARGs in soil floors and cow dung from rural Bangladesh households, reinforcing the value of environmental fecal matrices as resistance surveillance substrates.

Seasonal dynamics in WBE are a further analytical asset. Smith *et al.* (5) revealed distinct seasonal signatures for respiratory, enteric, and urinary viruses across a year-long wastewater sequencing program, demonstrating that metagenomics can track viral community composition shifts that precede clinical outbreak detection. Adapting this framework to livestock wastewater—where seasonal stocking densities, climate conditions, and disease calendars create predictable shedding windows—represents a high-priority research avenue. Challenges specific to veterinary WBE include variable dilution effects from rainfall and wash-down water in open-air farm drainage systems, high organic matter loads that inhibit nucleic acid extraction, and the absence of standardized concentration and extraction protocols validated across farm effluent matrices. Addressing these through internationally harmonized method validation will be a prerequisite for WBE to fulfil its potential as a routine veterinary surveillance tool (17, 18).

## 3 Applications of environmental surveillance

The disease-specific accounts that follow span viral, bacterial, and parasitological categories. The 15 specific diseases reviewed in this section were selected based on a structured four-pillar framework: (1) high global economic, production, and trade impact on domestic livestock and poultry; (2) substantial zoonotic risk or public health importance under a One Health mandate; (3) balanced representation across major taxonomic pathogen classes (viral, bacterial, and parasitological) and diverse transmission modalities (respiratory aerosols, waterborne dispersion, direct/fomite shedding, and vector-borne cycles); and (4) the availability of empirical peer-reviewed evidence evaluating environmental sample matrices. While these 15 diseases serve as representative models demonstrating distinct environmental surveillance modalities, the underlying surveillance principles apply across the broader spectrum of veterinary infectious diseases. Across all three categories, recurring analytical themes emerge: the suitability of different sample matrices for each pathogen, the comparative performance of detection methods under field versus laboratory conditions, the establishment of actionable detection thresholds, and the extent to which wastewater-based and other non-invasive approaches can contribute to herd-level monitoring. These dimensions are addressed within each disease discussion and synthesized comparatively in the subsequent subsections, summary tables, and Section 4.

### 3.1 Foot and mouth disease

Foot-and-mouth disease (FMD) is among the most economically damaging transboundary animal diseases globally, affecting all cloven-hoofed livestock and a broad range of wildlife. Transmission occurs principally through direct contact with infected animals and their secretions, but also via aerosol dispersion over distances of several kilometers under favorable meteorological conditions, and through contaminated fomites, animal products, and personnel—transmission pathways that render ES particularly informative (19). FMD virus (FMDV) is present in all secretions and excretions from acutely infected animals, including saliva, nasal fluid, exhaled air, feces, urine, semen, and milk, resulting in extensive environmental contamination during active outbreaks (19–22). Actionable environmental thresholds for FMDV are operationally defined: in free countries or containment zones, any confirmed RT-qPCR detection of FMDV RNA from milk collection centers, market transport stalls, or vehicle swabs prompts immediate statutory movement standstills, tracing of animal contacts, and mandatory virucidal disinfection with citric acid (0.2%) or sodium carbonate (4%) before premises clearance.

The environmental persistence of FMDV is governed by matrix type, pH, temperature, and relative humidity. Mielke and Garabed (23) synthesized available persistence literature and demonstrated that FMDV survival probability in water has a median of 28.5 days (range 11–30 days), whereas survival in biological fluids has a median of 5 days, and persistence under high-humidity, high-temperature conditions (86% RH, 37 °C) remains measurably positive beyond 24 h. Critically, FMDV is labile below pH 6.0, meaning acidic matrices—notably pig fecal slurry—inactivate the virus rapidly, whereas cattle fecal slurry maintains infectivity substantially longer. These matrix-specific survival characteristics have direct implications for sampling strategy: surfaces with neutral to alkaline pH, and matrices with low organic acid content, represent higher-probability detection substrates in field ES programs.

A pivotal contribution of ES to FMD surveillance has been the demonstration that FMDV RNA is detectable in the environment in the absence of clinically apparent infection. Colenutt *et al.* (24) collected samples from milk collection sites and households adjacent to infected premises in an FMD-endemic setting in Nepal, recovering FMDV RNA from locations with no animals exhibiting clinical signs. This finding is epidemiologically pivotal: it establishes that environmental positivity can precede or occur independently of clinical detection, extending the surveillance window and revealing cryptic transmission chains. The study also demonstrated that approximately 44% of FMDV transmission in modelled scenarios can be attributed to environmental contamination pathways, highlighting ES as integral to—rather than supplementary to—transmission network mapping (25). In an endemic field setting in Cameroon, viral RNA was detectable across soil, fomite, and air samples from infected cattle herds for up to 30 days post-outbreak onset, with detection rates varying substantially by matrix type and sampling proximity to infected animals, providing empirical support for matrix-stratified sampling protocols.

The probability of environmental FMDV detection is modulated by herd size, the proportion of acutely infected animals, shedding duration, and ambient climatic conditions—parameters that should inform sampling intensity and timing in surveillance protocols. Field data indicate that FMDV RNA has been recovered from cattle stalls up to 14 days after removal of infected calves, in urine at 39 days, and in soil for periods ranging from 3 days in summer to 28 days in autumn. Persistence on dry hay stored at 22 °C has been documented for up to 20 weeks, highlighting the biosecurity risk posed by contaminated fodder. Recovery efficiency is also matrix-dependent: when FMDV is dried onto surfaces, organic substrates such as hay and bran yield substantially higher recovery rates than inert materials including cotton wool and glass (24). These findings collectively argue for matrix-conscious sampling strategies that prioritize high-contact organic surfaces, feeding areas, and water troughs at animal aggregation points, particularly during and immediately following confirmed outbreaks.

### 3.2 Peste-des-petits ruminants

Peste des petits ruminants (PPR) is caused by PPRV (genus *Morbillivirus*, family *Paramyxoviridae*, species *Morbillivirus caprinae*) and is one of the most devastating viral diseases of small ruminants globally, with morbidity reaching up to 100% and case fatality rates up to 90% in naïve populations. Its targeted eradication by 2030—a goal jointly championed by the Food and Agriculture Organization of the United Nations (FAO) and the World Organisation for Animal Health (WOAH)—places particular demands on the sensitivity and reach of surveillance systems, especially in resource-constrained settings where clinical case detection is often delayed and animal-level sampling is logistically challenging.

Peste-des-petits ruminants virus is primarily transmitted via close direct contact and shed in nasal, ocular, oral, and fecal discharges, with consequent environmental contamination of housing, feeding areas, and shared water sources. Although the virus has limited stability outside the host, Brown *et al.* (26) demonstrated meaningful environmental detection rates in a longitudinal ES program in Plateau State, Nigeria: of 458 electrostatic dust cloth swabs collected from households, transhumance sites, and livestock markets over 7 months, 2.4% (*n* = 11) were positive for PPRV RNA, and an additional 1.3% (*n* = 6) positive for capripoxvirus DNA—including LSDV, sheeppox, and goatpox—from the same sampling events. This simultaneous multi-pathogen detection capacity is a key advantage of environmental swabbing at livestock markets, where diverse animals and disease pressures converge.

The analytical sensitivity underpinning environmental PPRV detection has been advanced by the development of a SYBR Green I-based reverse transcription-quantitative PCR (RT-qPCR) assay (27), which achieved a detection limit of 13.6 copies per reaction and an assay efficiency of 98.2%, representing a 3-log improvement in sensitivity over conventional RT-PCR. Critically, no cross-reactivity was observed with bluetongue virus, goatpox, or orf virus, confirming analytical specificity for field samples containing co-circulating small ruminant pathogens. Bataille *et al.* (28) further validated non-invasive fecal sampling approaches using RT-qPCR at a wildlife-livestock interface in Africa, demonstrating comparable sensitivity to invasive sampling methods and establishing the operational feasibility of eDNA-based PPRV surveillance in free-ranging ungulate populations. Together, these developments position ES as a genuine force multiplier for the global PPR eradication campaign, particularly in areas where wildlife-livestock interfaces and informal livestock markets sustain cryptic transmission.

### 3.3 Lumpy skin disease

Lumpy Skin Disease (LSD) is a rapidly expanding transboundary disease of cattle caused by LSDV (genus Capripoxvirus, family Poxviridae), which has spread dramatically from its African origins across the Middle East, Eastern Europe, South Asia, and into previously free regions of Southeast Asia over the past decade. Clinical disease is characterized by fever, generalized lymphadenopathy, and characteristic skin nodules, with profound economic consequences including reduced milk yield, weight loss, reproductive failure, hide damage, and high mortality in susceptible herds.

The enduring presence of LSDV in the environment is pronounced, posing difficulties for disease management. The virus exhibits remarkable stability, remaining active for long durations in infected tissues and dried scabs, which serve as primary sources of environmental contamination (29). This environmental resilience highlights the necessity of exhaustive decontamination, the use of effective virucidal agents such as sodium hypochlorite, formalin, and phenolic compounds for disinfection, and the proper disposal of infected carcasses and contaminated materials after an outbreak to decrease the environmental viral burden.

To monitor the environment for LSD, methods typically include collecting samples from surfaces, feeding areas, water sources, and possible insect carriers in and around impacted farms. Moreover, scabs and environmental swabs are gathered from areas suspected of contamination. Molecular techniques, especially real-time PCR (qPCR) that targets LSDV DNA, are frequently employed due to their high sensitivity and quick results, enabling the detection of viral genetic material even when the virus is not viable (30).

### 3.4 Avian influenza

Avian influenza (AI) viruses are type A influenza viruses (family *Orthomyxoviridae*) with segmented, negative-sense RNA genomes encoding 10–14 proteins. Pathotypically, they are classified as low pathogenic avian influenza (LPAI) or highly pathogenic avian influenza (HPAI) based on intravenous pathogenicity index and polybasic cleavage site characterization. The ongoing global spread of HPAI H5N1 clade 2.3.4.4b—with unprecedented poultry losses and documented mammalian spillover events (31)—has elevated AI to the highest tier of biosecurity concern and rendered ES an operational necessity (32). Environmental surveillance for AI spans a diverse matrix portfolio. Hood *et al.* (33), in an exhaustive systematic review of 407 peer-reviewed studies, catalogued ES applications across feces, mud, surface water, drinking water, feed, feathers, air, and contaminated market infrastructure including cages, cutting boards, and defeathering machines. This breadth of matrices reflects the virus’s capacity for high-titer fecal shedding by wild waterfowl, aerosol generation in confined poultry housing, and persistence in aquatic environments across a wide temperature range. A meta-analysis of wild bird AIV prevalence by Dupas *et al.* (34) across 2,851 studies from 1971 to 2023 confirmed that *Anatidae* exhibit an overall pooled prevalence of 6.19% (95% CI: 5.10–7.37%), with substantially higher rates in migratory species and those associated with freshwater habitats—ecological strata that should anchor ES monitoring site selection for HPAI early warning. In live bird markets (LBMs), environmental detection of H5 or H7 viral RNA from drinking troughs, cages, or defeathering machines triggers mandatory 24- to 48-h ‘market rest days’, total depopulation or stamping-out of unsold poultry, deep cleaning, and post-disinfection swabbing clearance before market reopening (33).

Wild aquatic birds, particularly *Anseriformes* (ducks, geese, swans) and *Charadriiformes* (gulls, terns), serve as the primary maintenance reservoir for LPAI viruses and the primary source population for HPAI spillover events (35). These birds shed high titers of AIV in feces and nasal secretions, directly contaminating aquatic environments that serve simultaneously as feeding grounds, staging areas, and virus amplification compartments. The infectivity of AIV in water is temperature-dependent: Brown *et al.* (36) demonstrated that viral persistence is maximized at low temperatures and neutral to slightly alkaline pH, with HPAI H5N1 remaining infectious for several weeks at 4 °C in surface water—conditions typical of northern wetland habitats during autumn migration.

The definitive demonstration that infectious HPAI can be recovered from surface water in the field came from Hubbard *et al.* (37), who isolated AIV from wetland water samples collected in April 2022 in Iowa, USA, during the major H5N1 HPAI outbreak, including confirmed HPAI H5N1 from one sampling site. The concentration protocol employed—sequential filtration, elution, and centrifugation steps achieving a median concentration factor of approximately 12,700-fold—is a methodological benchmark for environmental water-sample processing for infectious AI recovery. No AIV was isolated from May samples collected 6 weeks later, likely attributable to rising water temperatures, reduced waterfowl density, and diminished fecal loading—findings that underscore the narrow but critical temporal windows in which environmental water sampling for HPAI offers maximal yield.

Complementing water-based ES, Hostyn *et al.* (8) evaluated the Coriolis® μ liquid cyclone air sampler for HPAI H5N1 and NDV detection in both experimental and field settings. Air samples from outbreak poultry farms detected HPAI inside and outside barn structures, with outdoor aerial dispersion confirmed up to distances determined by wind direction and flock density. Critically, air sampling detected viral RNA beyond the period when tracheal and cloacal swabs from individual birds were positive, indicating that environmental air sampling extends the detection window and may capture residual aerosol contamination relevant to inter-farm biosecurity assessment. Chicken density was identified as a key determinant of aerial viral concentrations, with practical implications for risk-stratifying farms for targeted environmental monitoring.

### 3.5 Newcastle disease

Newcastle disease virus (NDV; genus Avulavirus, family *Paramyxoviridae*) causes one of the most economically impactful avian diseases globally, with virulent strains producing mortality rates approaching 100% in susceptible poultry. Unlike the matrix-specific survival characteristics of FMDV or HPAI, NDV exhibits a more generalized environmental persistence shaped primarily by temperature and moisture: at ambient room temperature, NDV remains detectable in poultry litter for up to 28 days, with notably extended survival under cool and humid conditions (38). Kinde *et al.* (38) examined NDV survival in compost and at ambient temperatures using both virus isolation and real-time RT-PCR, finding that RT-PCR retained sensitivity for RNA detection well beyond the window of infectious virus recovery—a distinction that has important operational implications: nucleic-acid positive environmental samples do not necessarily represent active infectious risk, and viability-linked assays should be considered when contamination assessments inform re-stocking decisions.

An important methodological advance for NDV environmental monitoring has been the evaluation of aerosol sampling. Hostyn *et al.* (8) used the Coriolis® micro air sampler to monitor NDV following live vaccine administration in broiler and layer farms, detecting vaccine-strain NDV in both indoor and outdoor air samples. Outdoor aerial dispersion extended up to 5 m from exhaust fans, with indoor viral concentrations correlating positively with bird density. Notably, air samples maintained high viral RNA concentrations even when individual bird swab positivity had declined, demonstrating that air sampling provides a more persistent and population-representative signal than swab-based approaches in high-density housing. Hostyn *et al.* (8) confirmed NDV RNA detectability in surface environmental samples using real-time RT-PCR, establishing a baseline molecular surveillance framework applicable across diverse farm settings. Together, these studies support a tiered ES approach for NDV: litter and fomite swabbing for pen-level contamination assessment, and air sampling for farm-level and inter-farm aerosol risk characterization.

### 3.6 African swine fever

African Swine Fever (ASF) is caused by ASFV—the sole member of the family Asfarviridae—and is the most consequential swine disease globally, with 100% case fatality rates in naïve domestic pig populations, no available vaccine, and a trajectory of intercontinental spread that has devastated pig production in Europe and Asia since 2007. The virus’s extraordinary environmental stability is a defining epidemiological feature: ASFV can remain infectious for weeks in blood, feces, and animal tissue, for months in chilled or frozen pork products, and for extended periods in soft tick vectors (*Ornithodoros* spp.) that sustain a sylvatic cycle in parts of Africa. Davies *et al.* (39) confirmed ASFV excretion in nasal, ocular, oral, and rectal secretions from experimentally infected pigs using the Georgia 2007/1 isolate, establishing the multi-route environmental contamination potential of the current pandemic strain.

The role of wild boar as the primary sylvatic reservoir and long-distance dispersal vector in Europe is well documented (40, 41). Wild boar carcasses represent particularly high-risk environmental substrates: infectious ASFV can be released from decomposing carcasses into surrounding soil and surface water, creating persistent contamination hotspots that can expose domestic pigs via pasture contact or scavenging. ES for ASF in endemic regions therefore encompasses: (i) systematic carcass searches and post-mortem sampling; (ii) soil and surface water sampling at carcass discovery sites and at wildlife-livestock interface zones; (iii) fomite swabbing of vehicles, equipment, and personnel entry points; and (iv) soft tick collection from pig housing in traditional African settings. All environmental sample types are screened by ASFV-specific PCR assays targeting conserved genomic regions, with confirmed positive samples subject to haemadsorption assay or virus isolation to confirm infectivity where biosafety infrastructure permits.

Geospatial integration has emerged as a critical force multiplier for ASF ES. Ito *et al.* (6) conducted a high-resolution spatial analysis of ASF in South Korea (2019–2021), demonstrating that spatial clustering of wild boar carcasses and positive environmental soil/water samples predicted subsequent domestic farm incursions. Rakotoarivony *et al.* (42) reinforced this dynamic using camera-trap methodology in Madagascar, showing that bushpig–domestic pig contact occurs primarily at shared water points and crop fields—identifying exact environmental nodes for targeted swabbing and eDNA monitoring. For ASFV, environmental qPCR positivity on livestock transport vehicles, lairage pens, or feed storage areas triggers immediate vehicle impoundment, quarantine, high-pressure thermal washing (>60 °C), and treatment with validated virucidal disinfectants (e.g., 2% sodium hydroxide or potassium peroxymonosulfate) with verification swabbing required prior to recertification.

### 3.7 Japanese encephalitis (JE)

Japanese Encephalitis (JE) is caused by JEV (genus *Orthoflavivirus*, family *Flaviviridae*) and constitutes the leading cause of viral encephalitis in Asia, responsible for approximately 68,000 clinical cases annually. In domestic animals, JEV causes reproductive failure—abortion and mummification in sows, neurological signs in piglets—and encephalitis in horses; pigs function as the primary vertebrate amplifier host, achieving high viraemia sufficient to infect naïve *Culex* mosquitoes (43). The JEV enzootic cycle is therefore tightly coupled to environmental determinants: *Culex* vector breeding is concentrated in irrigated rice paddies, effluent ponds, and stagnant water bodies, directly linking virus transmission intensity to hydrological and agricultural landscape features.

Traditional ES for JEV has been anchored in mosquito vector surveillance: trapping of *Culex* spp. using light traps or Mosquito Magnet® systems, followed by species identification, pooling (typically 5–25 individuals per pool), and JEV RNA detection by RT-PCR. Ritchie *et al.* (44) demonstrated operational feasibility of remote mosquito trap networks for JEV surveillance in the Torres Strait, Australia, a model subsequently expanded across endemic regions of Southeast Asia.

A paradigm-shifting development has been the application of WBE for JEV. Ahmed *et al.* (3) detected JEV RNA in municipal wastewater samples collected during an acute JEV outbreak in Australia, demonstrating that catchment-level wastewater provides a population-aggregated signal integrating pig shedding, human excretion from sub-clinical cases, and mosquito activity in a single sample. Ahmed *et al.* (3) extended this to piggery-specific effluent surveillance, showing that JEV RNA is reliably detectable in piggery wastewater during active transmission seasons, with RT-PCR sensitivity sufficient to capture low-viraemia shedding events. Critically, piggery WBE offers a herd-level signal independent of individual animal sampling logistics in large commercial operations, and can serve as an early warning trigger for intensified mosquito control and veterinary investigation. The integration of vector trap data, piggery WBE signals, and pig reproductive performance monitoring within a unified JEV ES framework represents the current state of the art and warrants formalization into national surveillance guidelines in JEV-endemic countries (summarized in Table 2).

**Table 2:** Summary of environmental sample matrices, detection methods, and field validation status for viral diseases of domestic animals.

Disease	Transmission route	Environmental persistence	Detection method	Optimal ES matrix	Field validation status	Key references
Foot-and-mouth disease	Direct contact, aerosol, fomites	Median 28.5 days in water; 5 days in biological fluids; 20 weeks on dry hay at 22 °C; labile below pH 6.0	RT-qPCR; virus isolation from organic substrates	Pen/floor swabs, expelled air, soil, water troughs	Field Validated — routine environmental swabbing in outbreak investigations; viability assays investigational	(20, 23, 24)
Peste des petits ruminants	Direct contact via secretions and excretions	Limited environmental stability; 2.4% positivity from 458 dust swabs over 7 months	SYBR Green I RT-qPCR (LOD 13.6 copies/rxn); non-invasive fecal RT-qPCR	Electrostatic dust cloths at livestock markets, communal water troughs	Field Validated — non-invasive dust/fecal swabbing validated at wildlife–livestock interfaces	(26–28)
Lumpy skin disease	Arthropod vectors, direct contact	Highly stable in dried scabs and infected tissues; months at ambient temperature	qPCR targeting LSDV DNA	Surface and equipment swabs, dried scabs	Field Validated — environmental surface and scab PCR used in endemic and outbreak monitoring	(26, 29, 30)
Avian influenza	Faecal shedding, nasal secretions, aerosol	Weeks at 4 °C in surface water (neutral–alkaline pH); temperature-dependent decay	Virus isolation (12,700 × concentration); RT-qPCR; Coriolis® air sampler	Surface water (wetlands); air samples (indoor housing); market infrastructure swabs	Operationally Established — wet market dust/water swabbing widely implemented; aerosol cyclone samplers field-validated	(8, 31, 33, 34, 37)
Newcastle disease	Respiratory discharges, feces	Up to 28 days in poultry litter at room temperature; extended under cool, humid conditions	Real-time RT-qPCR; Coriolis® air sampler for aerosol monitoring	Poultry litter, drinking water, air samples	Operationally Established — litter and air sampling validated for flock-level shed monitoring	(8, 38)
African swine fever	Direct contact, fomites, soft ticks (*Ornithodoros* spp.)	Weeks in blood/feces/tissue; months in chilled/frozen pork; persistent in decomposing carcasses	ASFV-specific qPCR; hemadsorption assay; GIS-integrated spatial risk mapping	Piggery effluent/lagoons, soil at carcass sites, feed, fomite swabs	Operationally Established — vehicle/facility swab qPCR and GIS carcass mapping operational globally	(6, 39–41, 76)
Japanese encephalitis	Mosquito-borne (*Culex* spp.)	Maintained through enzootic cycle; JEV RNA detectable in piggery effluent during transmission seasons	Mosquito pool RT-PCR; piggery wastewater RT-qPCR	Piggery wastewater, mosquito pools	Field Validated — piggery effluent WBE and vector trap RT-qPCR operational in outbreak zones	(3, 43, 44)

RT-qPCR, reverse transcription-quantitative PCR; qPCR, quantitative PCR; LOD, limit of detection; ES, environmental surveillance; WBE, wastewater-based epidemiology; GIS, geographic information systems. Validation classifications: Operationally Established (standardized in national/international surveillance programs); Field Validated (empirically confirmed in field/outbreak studies).

### 3.8 Salmonellosis

Salmonellosis, caused by diverse serovars of *Salmonella enterica*, represents one of the most epidemiologically complex bacterial zoonoses in animal production systems. Sub-clinical carriage is the norm rather than the exception: a substantial proportion of clinical-healthy cattle, pigs, and poultry shed *Salmonella* intermittently, creating persistent environmental reservoirs without generating diagnostic triggers from animal-level clinical surveillance. Environmental contamination spans bedding, floors, feed bins, water lines, dust, boot surfaces, and transport vehicles—matrices that collectively sustain within-farm cycling and enable between-farm spread via shared personnel and equipment.

*Salmonella* persistence in environmental matrices is broadly determined by moisture, temperature, pH, and organic matter content. In moist soil and manure under cool conditions, *Salmonella* can survive for several weeks to months (45). Wild birds—particularly gulls and passerines—have been identified as important secondary environmental sources, depositing *Salmonella* in outdoor farm areas and feed storage zones (46). This wildlife-livestock-environment interface complicates ES interpretation by creating background *Salmonella* positivity in outdoor matrices that may not originate from the production animals under surveillance.

The methodological toolkit for environmental *Salmonella* detection combines culture-based methods—selective enrichment on modified semi-solid Rappaport-Vassiliadis (MSRV) medium followed by xylose lysine deoxycholate (XLD) agar—with real-time PCR targeting the *invA* or *ttr* loci (47). Whole-genome sequencing (WGS) of environmental isolates has proven transformative for outbreak source attribution, tracing clonal strains across farms, transport crates, abattoirs, and retail products (45). Boot-sock swabbing of poultry house litter, drag swabs of manure channels, and sponge swabbing of transport vehicles represent validated non-invasive ES modalities that routinely detect herd/flock-level shedding in the absence of clinical signs. Regulatory thresholds for *Salmonella* are among the most clearly defined in veterinary environmental surveillance: under national and international poultry control programmes (such as EU Regulation (EC) No 2160/2003 and USDA-FSIS standards), detection of *Salmonella enterica* serovars Enteritidis or Typhimurium from pair-wise boot-sock or drag swabs in breeding or commercial layer flocks mandates immediate flock quarantine, prohibition of hatching egg movement, diversion of table eggs to pasteurisation, and comprehensive dry cleaning, washing, disinfection, and negative environmental clearance testing prior to chick placement (47, 48).

### 3.9 Paratuberculosis (Johne’s disease)

Paratuberculosis (Johne’s disease), caused by *Mycobacterium avium* subspecies *paratuberculosis* (MAP), is a chronic, progressive, and ultimately fatal granulomatous enteritis of ruminants. The disease’s insidious long incubation period—months to years between exposure and clinical onset—means that ES targeting environmental MAP reservoirs offers substantially earlier risk stratification than animal-level serology or individual fecal culture, which lack sensitivity in the pre-clinical stages of infection. Infected cattle shed MAP at escalating titers in feces as disease progresses, contaminating pastures, soil, communal water troughs, bedding, and milking areas—matrices that sustain viable MAP for extended periods owing to its robust mycobacterial cell wall.

*Mycobacterium avium* subspecies *paratuberculosis* environmental persistence is exceptional among bacterial pathogens in livestock settings. Singh *et al.* (50) pioneered environmental fecal sampling as a diagnostic and surveillance approach in Austrian cattle herds, demonstrating that pooled high-traffic area samples—collected from alleys, loafing areas, and calving pens—provided herd-level MAP status information comparable to individual animal testing at a fraction of the cost. MAP survives in cattle slurry for up to 15 months and in pasture soil for over a year, and shows marked resistance to composting and standard disinfectants (49). In Nepal, Singh (50) confirmed MAP detection by *IS900*-targeted qPCR in the faeces of dairy cattle, validating the *IS900* insertion sequence as the optimal molecular target for environmental samples due to its multi-copy nature and specificity for MAP.

Quantitative *IS900*-PCR is now the gold standard for environmental MAP detection, offering a 10-fold sensitivity advantage over liquid culture and delivering results in hours rather than the 8–16 weeks required for mycobacterial culture (50). Arango-Sabogal *et al.* (51) compared PCR and culture across environmental samples from dairy herds, demonstrating that pooled manure samples from cow alleys, loafing areas, and manure storage pits reliably reflect herd infection status, outperforming individual animal testing in cost-effectiveness. Quantitative thresholds for environmental MAP detection in herd slurry or composite manure (e.g., quantitative *IS900*-PCR Ct < 30, corresponding to >10^4 genomic equivalents per gram of manure) identify high-shedding ‘super-shedder’ herds that present severe transmission risks, enabling targeted segregation, calving-area sanitation, and herd biosecurity tiering; however, formal consensus cutoffs for herd-level freedom certification remain under active development through quantitative risk calibration.

### 3.10 Leptospirosis

Leptospirosis, caused by pathogenic *Leptospira* species, is the world’s most widespread zoonotic bacterial infection, yet its environmental epidemiology has historically been obscured by the extreme fastidiousness and slow growth of pathogenic leptospires in culture (52–54). Domestic animals—principally cattle, pigs, and dogs—serve as maintenance hosts for specific serovars (e.g., Hardjo in cattle, Pomona in pigs, Canicola in dogs), shedding billions of leptospires in urine directly into pasture, drinking water, and farm soils (53, 55).

Pathogenic *Leptospira* are obligate aerobes that thrive in warm (>20 °C), moist, neutral-to-alkaline (pH 7.0–7.4) environments, persisting for weeks to months in surface waters, mud, and waterlogged soils (56–58). They are rapidly inactivated by desiccation, pH < 6.0, direct solar ultraviolet radiation, and salinity > 1%, creating sharply defined environmental niches that correlate with disease hotspots. Biofilm formation on environmental substrates significantly extends leptospiral survival and confers resistance to chemical disinfectants (53, 56).

Ganoza *et al.* (59) demonstrated that pathogenic *Leptospira* DNA can be quantified from surface water samples in leptospirosis-endemic regions using quantitative PCR targeting the *lipL32* gene—a major outer membrane lipoprotein exclusive to pathogenic species. Casanovas-Massana *et al.* (60) provided definitive genetic proof of an environmental transmission pathway, matching *Leptospira interrogans* genotypes recovered from standing water and sewage with clinical isolates from infected individuals and animal reservoirs (57, 61). Sato *et al.* (62) advanced eDNA metabarcoding approaches that simultaneously detect pathogenic *Leptospira* and their mammalian host DNA from environmental water samples, offering a powerful tool for reservoir host attribution and environmental risk mapping at the livestock–wildlife interface.

### 3.11 Bovine tuberculosis (bTB)

Bovine tuberculosis (bTB), caused by *Mycobacterium bovis*, remains one of the most economically and politically contentious animal diseases in many countries, with the persistence of wildlife reservoir hosts—badgers (*Meles meles*) in the UK and Ireland, wild boar (*Sus scrofa*) in Continental Europe, white-tailed deer in North America, and Cape buffalo in sub-Saharan Africa—fundamentally constraining eradication through cattle-only test-and-slaughter strategies (63, 64). ES at the wildlife-livestock interface is therefore not an adjunct to bTB surveillance but a structural requirement for understanding and interrupting enzootic transmission cycles.

*Mycobacterium bovis* environmental persistence is facilitated by its waxy mycolic acid cell wall, which confers resistance to desiccation, acidic pH, and many disinfectants. Rodríguez-Hernández *et al.* (65) systematically reviewed *M. bovis* survival data, confirming that persistence is highly temperature-dependent and inversely related to average daily temperatures. The bacillus can survive for up to 88 days in soil and 58 days in water during cooler winter and spring months, whereas survival is reduced to approximately 7 days in the summer. This demonstrates that cool, moist substrates, particularly soil and faecal matter, serve as viable environmental reservoirs. Decomposing wildlife carcasses represent particularly concentrated point sources: infected badger or wild boar carcasses release viable *M. bovis* into surrounding soil and water as tissue integrity fails, creating persistent contamination hotspots that may remain infective for the entire duration of carcass decomposition (66).

A landmark advance in bTB ES was the first molecular detection of *M. bovis* in environmental samples from a French region with endemic bovine TB by Rodríguez-Hernández *et al.* (65). Using qPCR targeting the *IS6110* insertion sequence, the study detected *M. bovis* DNA in pasture soil and water samples from badger latrines at wildlife-cattle interface sites, establishing the operational feasibility of environmental molecular bTB surveillance. This approach bypasses the logistical, ethical, and economic challenges of live-trapping wildlife for test-and-cull programs by using non-invasively collected environmental samples as proxies for wildlife population infection status. Integration of environmental *M. bovis* detection data with granular wildlife movement and land-use datasets—through satellite tracking or camera-trap networks—represents the frontier of bTB ES, enabling precision targeting of intervention zones at the livestock-wildlife interface (summarized in Table 3).

**Table 3:** Summary of environmental sample matrices, detection methods, and field validation status for bacterial diseases of domestic animals.

Disease	Causative organism	Environmental persistence	Transmission & ES matrix	Detection method	Field validation status	Key references
Salmonellosis	*Salmonella enterica*	Weeks to months in moist soil and manure; biofilm formation extends persistence on farm surfaces	Fecal contamination; boot-sock swabs, drag swabs, feed, water lines, dust, transport vehicles	Selective enrichment culture (XLD agar) + real-time PCR; WGS for source attribution	Operationally Established — boot-sock and drag swab protocols underpin official statutory pre-harvest poultry monitoring globally	(46); (47); (45)
Paratuberculosis (Johne’s disease)	*Mycobacterium avium subsp. paratuberculosis* (MAP)	>1 year in manure, soil, water; mycolic cell wall confers exceptional environmental resilience	Faeco-oral; pooled fecal samples from alleys, loafing areas, calving pens, slurry pits	Quantitative *IS900*-qPCR (10 × sensitivity advantage over liquid culture); results in hours vs. 16 weeks	Operationally Established — pooled slurry/environmental fecal qPCR operational in national dairy herd health monitoring	(51); (50)
Leptospirosis	Pathogenic *Leptospira* spp.	Weeks to months in warm (>20 °C), moist, neutral–alkaline (pH 7.0–7.4) water/soil; biofilm-mediated persistence	Contaminated water/soil; surface water from ponds, streams, drainage ditches, flood-prone pastures	*lipL32*-qPCR; eDNA metabarcoding; 16S pre-rRNA transcript profiling; MAT for serovars	Field Validated — qPCR and eDNA metabarcoding field-tested in endemic wetlands; genomic transmission links established	(52, 59, 60, 62)
Bovine tuberculosis (bTB)	*Mycobacterium bovis*	Months in moist, cool, shaded conditions; waxy cell wall confers resistance to desiccation and disinfectants	Aerosol, faecal, urine contamination; soil/sediment at badger/wildlife latrines, carcass sites	*IS6110*-qPCR; culture + spoligotyping; environmental eDNA approaches	Field Validated — operational for wildlife reservoir latrine sampling; eDNA emerging for farm–wildlife interface	(63–65, 77)

qPCR, quantitative PCR; WGS, whole-genome sequencing; eDNA, environmental DNA; MAT, microscopic agglutination test; XLD, xylose lysine deoxycholate; ES, environmental surveillance. Validation classifications: Operationally Established (mandated in official surveillance regulations); Field Validated (confirmed under real-world agricultural/wildlife field conditions).

### 3.12 Coccidiosis

Coccidiosis, caused by species of the obligate intracellular apicomplexan protozoan genus *Eimeria*, is among the most economically devastating parasitic diseases of poultry and livestock globally, causing severe enteritis, malabsorption, diarrhoea, reduced feed conversion, and mortality. In domestic poultry (*Gallus gallus*), coccidiosis is caused by seven distinct pathogenic *Eimeria* species that exhibit specific intestinal predilection sites and varying virulence profiles (67–71): (1) *E. acervulina* (upper small intestine / duodenum; moderate pathogenicity producing characteristic white ladder-like transverse plaques and feed conversion loss); (2) *E. brunetti* (lower small intestine, caeca, and rectum; high pathogenicity causing severe coagulative mucosal necrosis); (3) *E. maxima* (middle small intestine / jejunum; moderate to high pathogenicity with petechial haemorrhages, distinctive large oocysts, and high immunogenicity); (4) *E. mitis* (lower small intestine; lower pathogenicity causing watery enteritis and growth depression); (5) *E. necatrix* (middle small intestine for severe destructive schizogony with caecal gametogony; highly pathogenic causing massive ballooning and haemorrhage); (6) *E. praecox* (upper small intestine; mild pathogenicity characterized by rapid prepatent development and reduced feed conversion); and (7) *E. tenella* (caeca; highly pathogenic causing severe haemorrhagic typhlitis, caecal cores, and high mortality in young broilers).

*Eimeria* oocysts are environmentally robust: sporulated oocysts can survive for months to years in cool, moist soil and litter, resisting desiccation and disinfectant pressures that readily inactivate most bacterial and viral pathogens. Environmental sampling for *Eimeria* focuses on litter, bedding, soil, and water in high-density housing—particularly calf pens and poultry houses in the first weeks of production when immunological naivety maximizes disease susceptibility. Waldenstedt (72) documented oocyst persistence patterns and risk factors in organic poultry systems, where outdoor access and accumulated pasture contamination create sustained high-challenge environments resistant to standard decontamination.

Methodologically, conventional McMaster flotation microscopy provides rapid oocyst quantification (oocysts per gram, OPG) but cannot differentiate these seven closely related *Eimeria* species due to overlapping oocyst morphometrics. Multiplex real-time PCR assays targeting the internal transcribed spacer (ITS-1 / ITS-2) regions or sequence-characterized amplified region (SCAR) markers directly from poultry litter and dust enable simultaneous species-specific identification and absolute quantification (67, 70, 71), revolutionizing environmental flock monitoring, vaccine take evaluation, and anticoccidial resistance management.

Environmental surveillance for *Eimeria* typically involves collecting samples from animal feces, bedding, soil, and surfaces within animal housing (such as poultry litter and calf pens) and on pastures (48). Commonly employed techniques include conventional microscopy for counting oocysts (e.g., McMaster technique) and molecular methods like quantitative PCR (qPCR) to detect and quantify oocysts and even differentiate *Eimeria* species from these environmental samples.

### 3.13 Fasciolosis

Fasciolosis, caused by *Fasciola hepatica* and the larger *Fasciola gigantica* in tropical regions, is a globally distributed helminthosis of ruminants and an important human zoonosis. It exerts substantial economic impact through liver condemnation at slaughter, reduced milk yield and live weight gain, impaired fertility, secondary bacterial infections via fluke-induced bile duct damage, and escalating anthelmintic resistance to triclabendazole—the cornerstone therapeutic—in sheep and cattle populations across Europe, Australasia, and South America. The disease’s complex indirect life cycle, obligatorily involving mud snails (*Lymnaea* truncatula for *F. hepatica*; *L. acuminata* for *F. gigantica*) as intermediate hosts, creates unique ES opportunities: the distribution and abundance of intermediate host snails and the density of infective metacercariae on pasture can be directly monitored as transmission risk indicators, without requiring animal-level clinical or pathological assessment.

Environmental contamination initiates when eggs excreted in faeces of infected hosts hatch in water, releasing free-swimming miracidia that infect snails within approximately 24–30 h of egg deposition. Within infected snails, asexual amplification generates large numbers of cercariae that encyst as metacercariae on aquatic vegetation—the infective stage for grazing ruminants. Metacercariae persist for weeks on moist pasture vegetation under cool conditions but are rapidly inactivated by desiccation and temperatures above 25 °C, making autumn and late winter the highest-risk transmission windows in temperate climates (73). The persistence of metacercariae on cut and ensiled forage has been documented, though survival is considerably reduced compared to pasture grass (73)—a practically important finding for farm-scale risk management.

Ciuca *et al.* (74) demonstrated a multi-environment eDNA approach for simultaneous quantification of *Fasciola* eggs and *Lymnaea* snail DNA from pasture soil and water samples, enabling fine-scale disease risk assessment across individual grazing paddocks. This eDNA-based framework represents a substantial methodological advance over traditional snail collection and manual metacercariae counting, offering higher throughput, reduced observer variability, and quantitative outputs directly amenable to risk modelling. Integration of eDNA-based ES data with satellite-derived wetness indices and snail habitat suitability models provides spatially explicit, near-real-time transmission risk maps—a framework particularly valuable for strategic targeted selective treatment programs aimed at reducing anthelmintic use.

The persistence of *Fasciola* in the environment is closely tied to the survival of its various life stages and its snail intermediate host. *Fasciola* eggs need water to develop and hatch, a process that can take from weeks to months depending on the temperature. The infected snail hosts, essential for parasite amplification, thrive in specific wet, muddy, and slow-moving water environments. Metacercariae, the infective stage, are highly resilient and can survive for several weeks on damp pasture vegetation, especially in cool and humid conditions, but are vulnerable to drying out and extremely high temperatures. The overall persistence of the parasite in a region depends on the continued presence of infected definitive hosts, suitable snail habitats, and favorable climatic conditions (73).

Environmental surveillance methods for fasciolosis typically involve mapping and surveying wet pasture areas, ditches, and ponds to identify and quantify populations of the intermediate host snails. Snails collected can be examined for *Fasciola* larval stages. Complementarily, pasture grass samples can be collected and examined microscopically for the presence of metacercariae, providing a direct measure of infective challenge (74). Faecal sedimentation tests in animals detect egg shedding, indirectly indicating environmental contamination. By actively monitoring the distribution and density of snail populations and the presence of infective metacercariae, veterinary authorities and farmers can predict periods of high transmission risk, identify high-risk grazing areas, and implement targeted control measures such as strategic deworming, fencing off snail habitats, or improving drainage to reduce snail populations and interrupt the parasite’s life cycle.

### 3.14 Babesiosis

Babesiosis, caused by intraerythrocytic protozoans of the genus *Babesia*, is an obligately tick-transmitted parasitic disease of global veterinary significance. *Babesia* bovis and *B. bigemina*—transmitted by *Rhipicephalus* (Boophilus) spp.—are responsible for bovine babesiosis across tropical and subtropical cattle-keeping regions, causing haemolytic anaemia, haemoglobinuria, and case fatality rates of 20–50% in naïve cattle. *Babesia* canis and B. gibsoni affect dogs; *B. caballi* and *Theileria equi* are relevant in horses. The obligate intracellular nature of *Babesia* parasites means that environmental persistence is exclusively mediated through tick vector biology: *Babesia* does not survive outside tick or mammalian host cells, and environmental detection is therefore synonymous with tick population and infection-status monitoring.

Ticks spend 80–90% of their life cycle off-host, in vegetation, leaf litter, and soil microhabitats, where they are accessible to ES without animal contact. Environmental tick surveillance employs flagging (dragging a white flannel cloth through vegetation) and CO₂ trapping to collect questing ticks, which are then morphologically identified to species, counted for population density estimates, and tested for *Babesia* infection using PCR. Rozej-Bielicka *et al.* (78) validated a high-resolution melting (HRM) PCR assay capable of detecting and differentiating multiple *Babesia* species infecting both humans and animals from pooled tick samples, demonstrating the applicability of HRM-PCR as a cost-effective, high-throughput environmental screening tool for *Babesia* serovar-level characterisation.

Landscape-scale ES of tick populations—integrating GPS-referenced tick density data with satellite-derived vegetation indices, temperature, and humidity data—enables predictive spatial risk modelling for babesiosis. Changes in tick geographic range driven by climate warming are already documented across Europe and North America, with ixodid ticks establishing in historically free regions and bringing attendant *Babesia* transmission risk. Environmental tick surveillance therefore serves both immediate operational biosecurity functions—informing acaricide treatment timing and dosing on affected farms—and longer-term climate adaptation planning in national animal health programmes (summarised in Table 4).

**Table 4:** Summary of environmental sample matrices, detection methods, and field validation status for parasitological diseases of domestic animals.

Disease	Causative agent	Transmission mode	ES matrix and methods	Quantitative environmental detection	Field validation status	Key references
Coccidiosis	*Eimeria* spp. (7 species in chickens)	Faeco-oral; oocyst ingestion from contaminated litter, soil, water	Faecal matter, bedding, dust, soil; McMaster microscopy and multiplex species-specific qPCR	Yes — multiplex qPCR enables absolute species-specific quantification; McMaster provides total OPG	Operationally Established — litter oocyst counts and qPCR routinely used in commercial poultry for anticoccidial efficacy monitoring	(48, 67, 70, 71)
Fasciolosis	*Fasciola hepatica*, *Fasciola gigantica*	Indirect life cycle via *Galba*/*Lymnaea* snail intermediate host; metacercariae on pasture	Mapping wet pasture areas; snail collection and dissection; sedimentation egg counts; water/pasture eDNA	Partial — snail density counts and sedimentation provide semi-quantitative indices; eDNA enables fine-scale risk mapping	Field Validated / Emerging — eDNA quantification of parasite and intermediate host DNA on pasture established in field trials	(73, 74)
Babesiosis	*Babesia* spp.	Tick-borne (*Ixodidae*: *Rhipicephalus*, *Ixodes* spp.)	Active tick collection by drag/flag sampling; morphological ID; high-resolution melting qPCR on tick pools	Partial — tick density estimation from standardized drag sampling; pooled tick infection rates reflect transmission pressure	Operationally Established for tick vector surveillance; direct environmental parasite quantification not applicable	(78)
Theileriosis	*Theileria* spp.	Tick-borne (*Ixodidae*: *Rhipicephalus*, *Hyalomma* spp.)	Active tick collection from vegetation (flagging/dragging) and cattle; PCR on tick homogenates	Partial — tick infestation rate and pooled tick PCR positivity rate reflect environmental infection pressure	Operationally Established for tick vector surveillance; direct environmental sporozoite quantification not applicable	(75)

OPG, oocysts per gram; qPCR, quantitative PCR; eDNA, environmental DNA; ES, environmental surveillance. Validation classifications: Operationally Established (standard operational protocol in veterinary diagnostics); Field Validated / Emerging (demonstrated in peer-reviewed agricultural field trials).

The survival of *Babesia* in nature relies entirely on the life cycle and persistence of its tick vectors, which act as both biological hosts and environmental reservoirs. Ticks spend much of their life cycle off the host, in environments like vegetation, leaf litter, and soil, where they develop from eggs into larvae, nymphs, and adults. Methods for environmental surveillance of babesiosis mainly include actively collecting ticks from the environment. These ticks are then identified by species through morphological examination, counted to estimate their population density, and can be pooled and tested for *Babesia* DNA using sensitive molecular techniques such as PCR (78).

### 3.15 Theileriosis

Theileriosis encompasses a spectrum of tick-transmitted diseases caused by obligate intracellular protozoans of the genus *Theileria*, with *T. parva* (East Coast fever; vector *Rhipicephalus appendiculatus*) and *T. annulata* (tropical theileriosis; vector *Hyalomma* spp.) representing the most economically impactful species in sub-Saharan Africa and North Africa/Asia, respectively. East Coast fever alone is responsible for estimated annual losses of over USD 300 million in sub-Saharan Africa, with case fatality rates in susceptible cattle approaching 100% without treatment. Unlike babesiosis, the *Theileria* life cycle includes a unique stage of host lymphocyte transformation, driving lymphoproliferative disease rather than purely haemolytic anaemia.

As obligate intracellular organisms, *Theileria* spp. are entirely dependent on tick vectors for environmental persistence and transmission. *T. parva* undergoes transstadial—but not transovarial—transmission in *Rhipicephalus appendiculatus*, meaning that larval and nymphal ticks must feed on infected buffalo or cattle to acquire infection, and the subsequent nymphal or adult stage transmits to naïve cattle. This stage-dependent acquisition biology means that the epidemiological risk from a given tick population depends not only on density but on the infection prevalence across tick life stages—a nuance that environmental tick-based surveillance must capture.

Environmental surveillance for theileriosis employs active tick collection from vegetation and cattle—using flagging, animal-associated collection, and dip-tank tick submission—followed by species identification and PCR-based *Theileria* detection. Kirvar *et al.* (75) developed PCR assays using *Tams1* gene sequences for *T. annulata* detection in cattle and ticks, establishing the molecular diagnostic basis for environmental tick-based surveillance. More recently, high-throughput next-generation sequencing of pooled tick homogenates has enabled simultaneous detection of multiple *Theileria* species and co-infecting tick-borne pathogens from environmental samples, providing richer epidemiological data per sampling effort. Longitudinal ES monitoring of tick infection rates across seasons and agro-ecological zones informs strategic acaricide application timing and the targeting of prophylactic immunization with the infection-and-treatment method for East Coast fever in high-risk zones (summarized in Table 4).

*Theileria* parasites are obligate intracellular organisms that are exclusively spread by certain hard tick species, such as *Rhipicephalus appendiculatus* for *T. parva* and *Hyalomma* spp. for *T. annulata*. Unlike babesiosis, these parasites cannot survive independently in the environment; their survival and spread are entirely dependent on their tick vectors. Consequently, monitoring for theileriosis involves assessing the presence, population, and infection status of these vector ticks in grazing areas and other suitable environments. *Theileria* parasites persist in the environment through the survival and life cycle of their tick carriers. Surveillance methods for theileriosis mainly include actively collecting ticks and testing them for *Theileria* DNA using highly sensitive molecular techniques like PCR (75) (summarized in Table 4).

## 4 Limitations and implementation challenges

Environmental surveillance holds transformative potential, but its responsible application requires clear-eyed acknowledgement of substantial interpretive and methodological limitations. Chief among these is the critical diagnostic distinction between molecular nucleic acid detection and viable, infectious pathogen presence. PCR-based assays amplify target DNA or RNA irrespective of organism viability, potentially yielding positive signals from degraded viral fragments, non-viable bacterial cells, or inactivated oocysts lingering in soil, manure, or equipment long after active infection has ceased. To bridge this viability gap, several viability-linked molecular techniques have emerged, including propidium monoazide (PMA) or ethidium monoazide (EMA) pre-treatment viability-qPCR (v-qPCR), RNA-to-DNA integrity ratios, and precursor ribosomal RNA (pre-rRNA) transcript decay profiling. However, as synthesized in Table 5, viability-linked assays face major matrix-specific barriers under field conditions: high sample turbidity and particulate matter obstruct the light photoactivation required for PMA cross-linking; complex organic matrices can exert cytotoxic effects that non-specifically compromise cell membrane integrity; and validation across complex veterinary matrices (slurry, soil, dust) remains limited compared to clean clinical or drinking water matrices. Consequently, culture isolation and *in vivo* bioassays remain the gold standards for establishing infectious risk, though their prolonged turnaround times and biosafety containment demands limit their routine field deployment.

**Table 5:** Current status of viability-linked assays and validation gaps for environmental pathogen surveillance across domestic animal diseases.

**Pathogen / disease group**	**Primary environmental matrix**	**Standard molecular target**	**Viability assay approach**	**Validation status in environmental matrices**	**Operational limitations and key validation gaps**
Avian Influenza Virus (HPAI H5N1)	Surface water, wetland sediment, indoor air	M gene / HA gene RT-qPCR	Embryonated chicken egg (ECE) inoculation; v-RT-qPCR (PMAxx / EMA)	Field Validated for egg culture; v-RT-qPCR investigational	Egg inoculation requires BSL-3 biocontainment and 3–7 days turnaround; PMAxx dye penetration is variable across enveloped viral capsids in turbid wetland water.
Foot-and-Mouth Disease Virus (FMDV)	Barn floor swabs, hay, slurry, expelled aerosols	3D / 5’ UTR RT-qPCR	Primary bovine thyroid (BTY) / IB-RS-2 cell culture; PMA-RT-qPCR	Investigational / Laboratory Phase	Primary cell isolation from fomites has very low sensitivity; slurry extracts exhibit severe cytotoxicity to cell monolayers; v-RT-qPCR lacks field matrix validation.
African Swine Fever Virus (ASFV)	Soil at carcass sites, piggery effluent, transport fomites	VP72 (B646L) gene qPCR	Haemadsorption (HAD) assay in primary alveolar macrophages (PAMs); PMAxx-qPCR	Investigational in Environmental Matrices	PAM culture requires fresh porcine lung tissue and primary cell facilities; low recovery efficiency from desiccated environmental substrates; HAD assay requires 5–7 days.
*Salmonella enterica*	Boot-sock swabs, poultry litter, dust, water lines	*invA* / *ttr* gene qPCR	Selective pre-enrichment culture (ISO 6579); PMA-qPCR; RNA-to-DNA integrity ratio	Operationally Established for culture; Field Validated for PMA-qPCR	Selective enrichment culture remains regulatory gold standard; PMA-qPCR experiences partial dye exclusion in high-fat/protein poultry litter and dead-cell signal carryover.
*Mycobacterium avium subsp. paratuberculosis* (MAP)	Dairy herd slurry, manure lagoons, pen bedding	*IS900* / F57 qPCR	Liquid culture (MGIT / TREK-ESP; 8–16 weeks); phage amplification assay; PMA-qPCR	Investigational for rapid viability; Culture validated but prolonged	Liquid culture takes 8–16 weeks and suffers high fungal/bacterial overgrowth; thick waxy mycolic cell wall impedes PMA dye penetration into compromised cells.
Pathogenic *Leptospira* spp.	Agricultural drainage water, mud, flooded pasture	*lipL32* qPCR, 16S rRNA	EMJH liquid culture; viability qPCR (PMAxx); 16S pre-rRNA decay profiling	Emerging / Investigational in Environmental Water	Slow culture (weeks to months) is rapidly overgrown by saprophytic leptospires; aquatic humic and fulvic acids interfere with PMA photoactivation and PCR amplification.
*Eimeria* spp. (Coccidiosis)	Poultry house litter, feces, soil	ITS-1 / SCAR qPCR	*In vitro* sporulation assay + microscopic counting; bioassay; excystation viability staining	Operationally Established for sporulation; v-PCR experimental	Microscopic sporulation scoring is labor-intensive and lacks species differentiation; rigid oocyst wall resists dye uptake until mechanical or enzymatic excystation.

v-qPCR, viability quantitative PCR; PMA, propidium monoazide; EMA, ethidium monoazide; RT-qPCR, reverse transcription-quantitative PCR; BSL, biosafety level; ECE, embryonated chicken eggs; PAM, porcine alveolar macrophages; MGIT, mycobacteria growth indicator tube; EMJH, Ellinghausen-McCullough-Johnson-Harris medium; OPG, oocysts per gram; ES, environmental surveillance.

Matrix-specific inhibition represents a second major analytical challenge. Faecal, soil, and manure matrices contain humic acids, bile salts, and complex organic compounds that co-purify with nucleic acids and inhibit PCR amplification, inflating false-negative rates in ways that are difficult to quantify without systematic internal amplification controls (11). Recovery efficiencies vary substantially across extraction protocols, concentration methods, and matrix types, yet few published ES studies report full performance metrics—detection limits, extraction efficiency controls, and inhibition assay results—that would permit meaningful inter-study comparison. The absence of harmonized, matrix-validated standard operating protocols across national reference laboratories constitutes a systemic weakness that limits evidence synthesis and regulatory uptake.

Sampling design and statistical representativeness present additional constraints: point sampling in heterogeneous environments (e.g., a single water sample from a 10-hectare wetland, or a single litter swab from a 50,000-bird broiler house) may systematically misrepresent environmental contamination burden due to spatial aggregation, dilution effects, and pathogen clumping. Spatial and temporal sampling frameworks informed by geostatistical modelling and pathogen persistence kinetics are needed to ensure that detection probabilities are quantified rather than assumed.

At the operational and governance level, the translation of environmental signals into actionable veterinary decisions requires predefined quantitative response thresholds and standardized regulatory frameworks. As illustrated in the disease-specific reviews, operational thresholds exist for established programs—such as mandatory flock restrictions and cleaning clearance following *Salmonella* detection in poultry boot-sock swabs (EU Regulation (EC) No 2160/2003), or live bird market rest days upon environmental H5/H7 avian influenza RNA detection. However, for pathogens such as MAP in dairy slurry or subclinical JEV in piggery wastewater, formal regulatory thresholds remain undefined. Developing actionable thresholds requires: (1) establishing quantitative baseline shed loads across seasons and production types; (2) calibrating environmental qPCR cycle threshold values and copy numbers against herd-level clinical attack rates using receiver operating characteristic (ROC) curves; and (3) applying quantitative microbial risk assessment (QMRA) to determine infectious dose thresholds that trigger graded veterinary interventions (e.g., advisory alerts, movement standstills, mandatory vector abatement, or premises disinfection).

A further structural constraint is the gap between laboratory-validated and field-validated performance. Most published ES methods have been developed and evaluated under controlled laboratory conditions, and their diagnostic sensitivity and specificity in complex field matrices remain insufficiently characterized. Large-scale ring trials and inter-laboratory validation studies across diverse environmental matrices and epidemiological contexts are urgently required to establish standardized performance metrics and benchmark new technologies against existing gold standards.

Broader adoption will also necessitate data-management systems that connect environmental findings to veterinary reporting systems, outbreak investigation processes, and policy-making. New technologies like portable sequencing, biosensor platforms, and AI/ML-assisted pattern detection could enhance the speed and interpretation of data by identifying spatiotemporal clusters, anomaly signals, and trends specific to matrices across repeated environmental sampling. Nonetheless, these systems will require field validation, workforce training, and appropriate governance before they can be routinely used in surveillance.

## 5 Conclusion

Environmental surveillance has matured from a research curiosity into an operationally relevant pillar of infectious disease monitoring in domestic animal populations, and the evidence reviewed here demonstrates its distinctive and irreplaceable epidemiological contributions. Across 15 disease contexts—from FMDV aerosol persistence in endemic settings to eDNA detection of *Fasciola* metacercariae on pasture; from WBE for JEV in piggery effluent to molecular bTB detection at wildlife-cattle interfaces—ES has consistently demonstrated the capacity to detect transmission before clinical cases accumulate, to characterize contamination hotspots with spatial granularity impossible through animal-level surveillance alone, and to integrate across human, animal, and environmental health data streams in the spirit of the One Health framework.

Matrix selection remains the foundational decision in ES design, and our comparative analysis across diseases reveals consistent patterns: water bodies and aquatic sediments are optimal for AIV, *Leptospira*, and JEV; pooled environmental faeces and litter excel for MAP, *Salmonella*, *Eimeria*, and enteric viruses; air sampling adds unique detection windows for respiratory pathogens including HPAI and NDV; tick-based environmental sampling is indispensable for babesiosis and theileriosis; and fomite and dust cloth swabbing provides cost-effective, simultaneous multi-pathogen signals at livestock markets and household interfaces.

Realizing the full promise of ES within routine veterinary surveillance requires progress on four interconnected fronts: (i) harmonized, matrix-validated standard operating procedures that enable inter-laboratory and cross-national comparability; (ii) viability-linked molecular assays that distinguish infectious risk from nucleic acid residue; (iii) evidence-based decision thresholds that translate environmental signals into defined management actions; and (iv) data integration infrastructure that connects ES outputs to national veterinary reporting systems, outbreak investigation databases, and risk-based surveillance programs. Wastewater-based epidemiology, which has demonstrated dramatic utility in human health surveillance, is an underexploited frontier in veterinary ES that deserves structured research investment, particularly for intensively-reared species and zoonotic pathogens where population-level monitoring without animal handling is a practical imperative. With these foundations in place, ES is poised to transition from an investigational adjunct into a routine surveillance modality that, in concert with animal-level and genomic surveillance, forms the sensing infrastructure for a genuinely integrated One Health disease intelligence system.
